# A Rare and Atypical Manifestation of Intraosseous Hemangioma in the Zygomatic Bone

**DOI:** 10.3390/diagnostics15151979

**Published:** 2025-08-07

**Authors:** Evagelos Kalfarentzos, Efthymios Mavrakos, Kamil Nelke, Andreas Kouroumalis, Gerasimos Moschonas, Argyro Mellou, Anastasia Therapontos, Christos Perisanidis

**Affiliations:** 1 Oral and Maxilofacial Surgery University Clinic, Dental School, National and Kapodistrian University of Athens, Evangelismos General Hospital, 10676 Athens, Greece; evakalf@dent.uoa.gr (E.K.); andrewkouroumalis@yahoo.gr (A.K.); ger.92.mos@gmail.com (G.M.); cperis@dent.uoa.gr (C.P.); 2 Maxillo-Facial Surgery Ward, EMC Hospital, Pilczycka 144, 54-144 Wrocław, Poland; 3 Department of Pathology, Evangelismos General Hospital, 10676 Athens, Greece; argiro.mellou@gmail.com (A.M.); anactasiath98@gmail.com (A.T.)

**Keywords:** intraosseous hemangioma, zygomatic bone, computerized tomography, tumor resection, radiology, bone lesion

## Abstract

Intraosseous hemangiomas (IH) are rare intrabony lesions that represent less than 1% of intraosseous tumors. IH are mostly seen in the axial skeleton and skull. Most commonly, the frontal bone, zygomatic, sphenoid, maxilla, ethmoid, and lacrimal bone can manifest IH. Currently, IH is classified as a developmental condition of endothelial origin. According to WHO, the five histological types of IH are cavernous, capillary, epithelioid, histiocytoid, and sclerosing. IH of the zygoma is an extremely rare condition with female predominance. A systematic review recently estimated that there were 78 cases published in the literature until 2023. The lesion is usually asymptomatic and presents with a gradually deteriorating deformity of the malar area, and the patient might be able to recall a history of trauma. Numbness due to involvement of the infraorbital nerve might also be present; however, atypical skin and bone sensations might also occur. Other symptoms include painful swelling, bone asymmetry, skin irritation, sinus pressure, paresthesia, diplopia, enophthalmos, or atypical neuralgia. A bony lesion with a trabecular pattern in a radiating formation (sunburst pattern) or a multilocal lytic lesion pattern created by the multiple cavernous spaces (honeycomb pattern) is commonly observed during radiologic evaluation. We present a rare case of IH of the zygoma in a 65-year-old generally healthy woman. A cyst-like bone tumor was revealed from the CT scan, which made preoperative biopsy of the lesion problematic. A careful radiological diagnostic differentiation of the lesion should always be conducted in such cases to outline a safe surgical plan and possible alternatives if needed. The patient underwent total tumor resection in the operating room, and the defect was reconstructed with the use of a titanium mesh and a synthetic hydroxyapatite bone graft based on a 3D surgical guide printed model.

**Figure 1:**
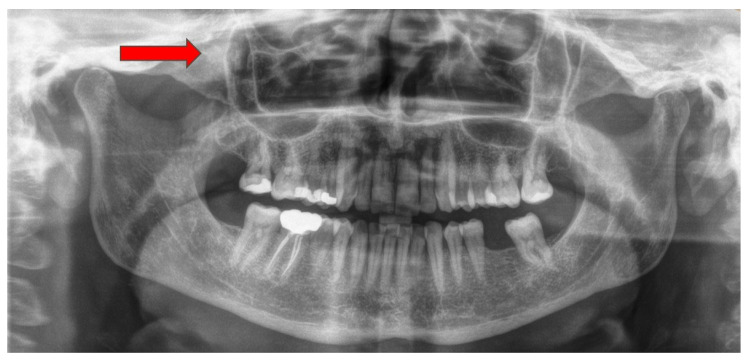
Many bone lesions can be easily detected on a routine radiological screening with a panoramic radiograph. In some alarming cases of atypical clinical signs and symptoms, followed by a bone area that is less likely to be estimated in a panoramic X-ray, a more detailed radiological investigation is necessary. A 65-year-old lady was consulted because of a mass in the right infraorbital rim and zygomatic area that progressively increased in size during the last 12 months. The patient complained of intermittent pain in the area that was alleviated with the intake of acetaminophen. Pain and atypical swelling along with numbness were present in the right zygomatic–maxillary area (red arrow). The patient also reported a burning sensation in the right malar area adjacent to the zygomatic bone. She had previously visited a dermatologist because of the assumption that a lesion similar to a cyst that she had removed years ago from the same site had reoccurred, but dermatologic consultation was without findings. During standard dental and surgical examination, a palpable hard mass was present, covered by bone, and had clear margins that separated it from the adjacent soft tissue. The mass was painless on palpation with no signs of inflammation or pus expression. The patient was healthy without any bone-related diseases or usage of medications such as bisphosphonates or other bone-related medications. Trauma and infection in this area were excluded from the patient’s history. Patient’s blood work was without any abnormalities, and the blood panel for calcium-phosphate disturbances did not reveal anything atypical. On a routine panoramic X-ray, an ovalar radiopacity in the right zygomatic bone is appreciable (red arrow). The lesion’s nonspecific radiological characteristics and atypical site made it hard to evaluate without further radiological examination. A more detailed diagnostic imaging was ordered because of the complex clinical presentation of the patient.

**Figure 2:**
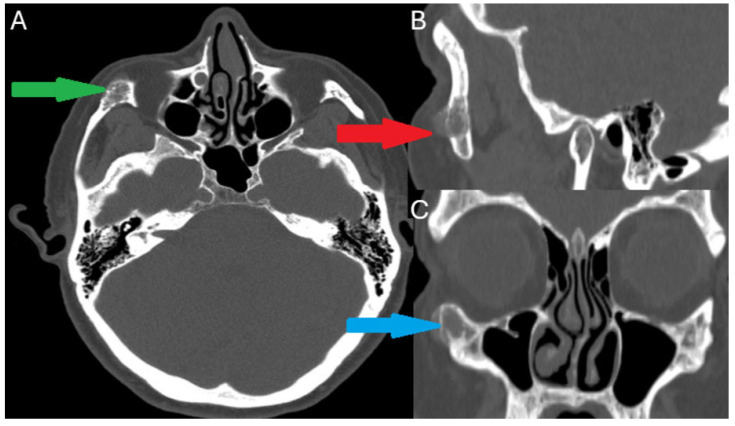
Because of atypical bone symptoms, more improved diagnostics were performed. Initial CT scan with contrast enhancement (GE Medical Systems, Optima CT 540, Bengaluru, India). In the axial view (green arrow), a radiolucent lesion of low density is seen within the body of the right zygomatic bone. The lesion has dimensions of 1.5 × 1.0 cm and a trabecular appearance (**A**). Within the lesion, radiopaque spikes are seen radiating outwards of the lesion following a sunburst pattern. On the lateral view, the red arrow points out that the lesion is well circumscribed, although the cortical layer of the zygomatic bone is eroded (**B**). There are no signs of periosteal reaction. Some atypical bone swelling and elevation with a mixed radiolucent-radiopaque lesion on the border of the inferior orbital rim and the zygomatico-maxillary junction is noted (blue arrow) on the coronal view (**C**). Similar radiologic findings in this area should be differentiated from fibrous dysplasia, osteochondroma, meningioma, osteogenic sarcoma, osteomyelitis, atypical bone inflammation, and multiple myeloma (MM). Based on the radiological findings, the suspicion of a benign intraosseous lesion was high. Because of the atypical location, symptoms, and radiological appearance, a decision for an excisional biopsy was made. We avoided the performance of a fine needle aspiration biopsy (FNAB), since the bone that covers the lesion makes it hard to penetrate, and the possibility of bleeding was high [1]. This decision was made because there were no worrisome symptoms like fatigue, weight loss, or anemia. On the other hand, because of the radiological appearance, authors initially differentiated the lesion between ossifying fibroma (OsF), CGCG (central giant cell granuloma), or an intraosseous hemangioma (IH). The suspicion for IH was high because of the characteristic pattern in CT scans. A well-circumscribed lesion with clear borders and a radiating trabecular appearance following a ‘’sunburst ‘’pattern can be pathognomonic for IH [2]. MRI can add valuable information regarding soft tissue involvement and is superior to a CT scan for the evaluation of vascular lesions [3]. Some studies reported that on MRI, IH are typically seen as hyperintense lesions on T2 images and hypointense or hyperintense on T1 images depending on the size of the lesion [3,4]. Generally, in radiological examination, after contrast, the lesion is more visible; however, small lesions are seen as hyperintense while larger lesions are hypointense on T1-weighted images [3,4]. The ratio of red and yellow (fatty) marrow within the lesion influences the MRI appearance of IH [2]. It is always worth estimating the periosteal condition and the status of adjacent bones. The absence of periosteal reaction made osteogenic sarcoma a less possible outcome than other entities. Intraosseous meningioma was also excluded because of the atypical site of that lesion and the absence of soft tissues. Fibrous dysplasia has a ground-glass appearance, and the lesion does not follow a radiating pattern. Osteochondroma was also excluded because of the irregular shape that it often has and the absence of corticomedullary continuity, a projection from the main bone which is characteristic of osteochondromas. Finally, there were no multiple lesions nor osteoporotic bone that would suggest multiple myeloma.

**Figure 3:**
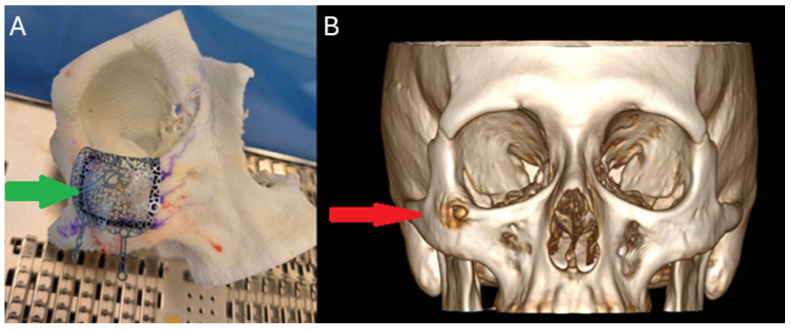
Treatment options for IH are surgical excision, sclerotherapy, radiation, or cryotherapy of the tumor [5]. During preparation for surgery authors used a 3D-printed model (**A**) (Form 3B, Formlabs, Somerville, MA, USA) to mark the surgical margins and establish the best approach for the patient. The creation of the 3D acrylic model was based on the location of the lesion (red arrow) in the patient’s initial CT-3D reformation (**B**). Intraoperatively, we used a titanium mesh (green arrow) and bent it accordingly to mimic the patient’s anatomical contours. It is worth remembering about possible alternative approaches and their indications, different than an open surgery. Radiation is rarely recommended because there is a risk of malignant transformation, and it might potentially prohibit bone growth in children [5]. Indications for radiation include increased tumor size and surgical inaccessibility for dissection. Sclerotic agents, ethanol or sodium morrhuate, can be injected into the lesion and induce an inflammatory response that shrinks the tumor or can significantly reduce its size. Sclerotherapy results in the elimination of blood vessels, but if blood flow is high, sclerotic agents might fail, compromising the results of the procedure. On the other hand, cryotherapy could also be used, but it can damage the innervation of adjacent tissues. In some cases, the lesions can be dealt with observation alone; however, some atypical swelling, pain, and other cosmetic issues for the patient are an indication for an open surgery [6]. In each case, maintaining good bone symmetry and facial features, along with a cosmetic result, is crucial given the benign nature of the tumor. Mostly, radical surgical removal is mandatory to avoid any lesion progression or recurrence. Each approach should be planned to achieve clear margins and to reduce any possible relapse of the disease. Complete surgical excision with en bloc removal of the tumor is the treatment of choice for most cases of IH. Partial excision is also an option for asymptomatic patients operated for cosmetic reasons purely, but there is a possibility for recurrence [7]. Reconstruction of the deficit can be performed by using autologous bone graft from the calvarial bone, rib, hydroxyapatite, silicone, or pedicled flaps from the infratemporal fossa, or with the use of patient-specific implants (PSI) or other solutions. The CT scan can also be used after mirroring the unaffected side to construct a perfect mirrored PSI.

**Figure 4:**
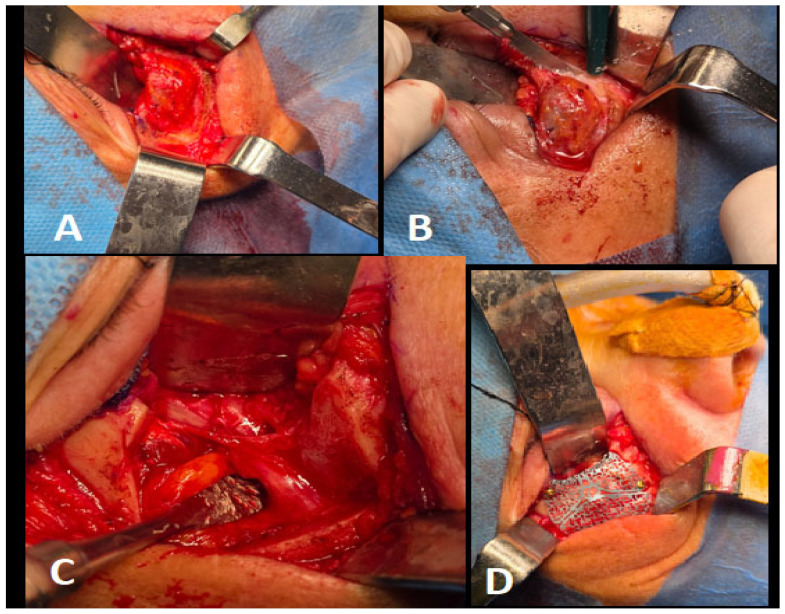
Surgery was performed under general anesthesia via nasotracheal intubation for complete removal of the tumor. To protect the eye during surgery, a tarsorrhaphy suture (Vicryl Rapid 4-0, Ethicon, Raritan, NJ, USA) was applied. A lower lid crease incision was made to expose the zygomatic bone, inferior orbital rim, and malar prominence, fully revealing the tumor (**A**). The orbital floor was elevated in a subperiosteal plane and gently retracted using a standard surgical spatula (Schwert Minnesota 2460, Tuttlingen, Germany), allowing protection of the orbital contents. Tumor excision was carried out through osteotomies as planned preoperatively, utilizing a surgical reciprocating saw (S8-R, Implantmed-W&H, Burnoos, Austria) (**B**). Intraoperative bleeding was minimal. En bloc removal of the tumor was successfully achieved with clear bone margins and preservation of the infraorbital nerve (**C**). The bone deficit measured 2.5 × 1 × 0.5 cm and was surrounded by healthy bone and an intact maxillary sinus membrane without perforation. Reconstruction was accomplished using synthetic hydroxyapatite alloplastic bone (OsteoSparx, IsoTis, Irvine, CA, USA), gently layered into the defect cavity until filled. A pre-contoured small orbital titanium mesh (Modus 2, Medartis, Basel, Switzerland) was positioned to protect the graft and stabilize orbital contents. The mesh was secured with four 1.5 mm screws (Modus 1.5, Medartis, Basel, Switzerland) (**D**). After inspecting the surgical field for hemorrhage and performing thorough irrigation, the wound was closed in layers. Symmetry of eye position was verified following removal of the protective tarsorrhaphy, and a forced duction test was performed to rule out entrapment of the inferior rectus muscle. The patient recovered uneventfully from anesthesia and was transferred to the ward.

**Figure 5:**
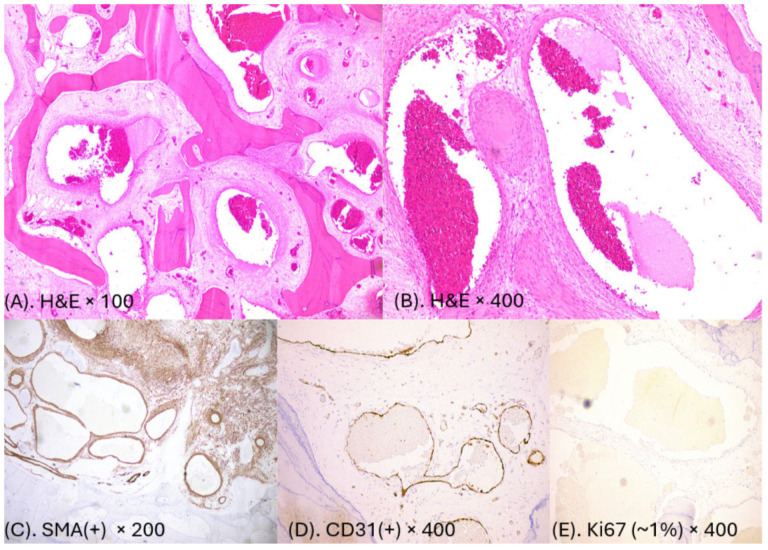
The histopathological examination revealed a lesion that in H&E (hematoxylin and eosin)-stained tissue sections consisted of multiple large/dilated, cystic, thin-walled, blood-filled spaces/vessels lined by a single layer of flat, cytologically banal–no atypical endothelial cells and separated by scant connective tissue stroma (**A**,**B**). The vessels permeated the marrow and surround pre-existing trabeculae. Immunohistochemistry showed that neoplastic cells were positive for a-SMA (actin) (**C**) and CD31 (**D**). The other markers (CD34, ERG, FLI1, D240, ER, PGR, DESMIN, AE1/AE3, and HHV8) were negative. The marker of cell proliferation (Ki67) was measured at 1%—very low—and enhanced the benign nature of the lesion (**E**). The final diagnosis was hemangioma of the bone/cavernous hemangioma [WHO 5th edition Head and Neck tumors//Soft Tissue tumors//Vascular tumors//Hemangioma, ICD-O 9121/0 Cavernous Hemangioma] [8]. The tumor was resected within microscopically healthy margins (R0 resection). Gathered IH microscopic specimens are the same as in known literature, and no additional features or extracellular manifestations were noted in the author’s study [4,5].

**Figure 6:**
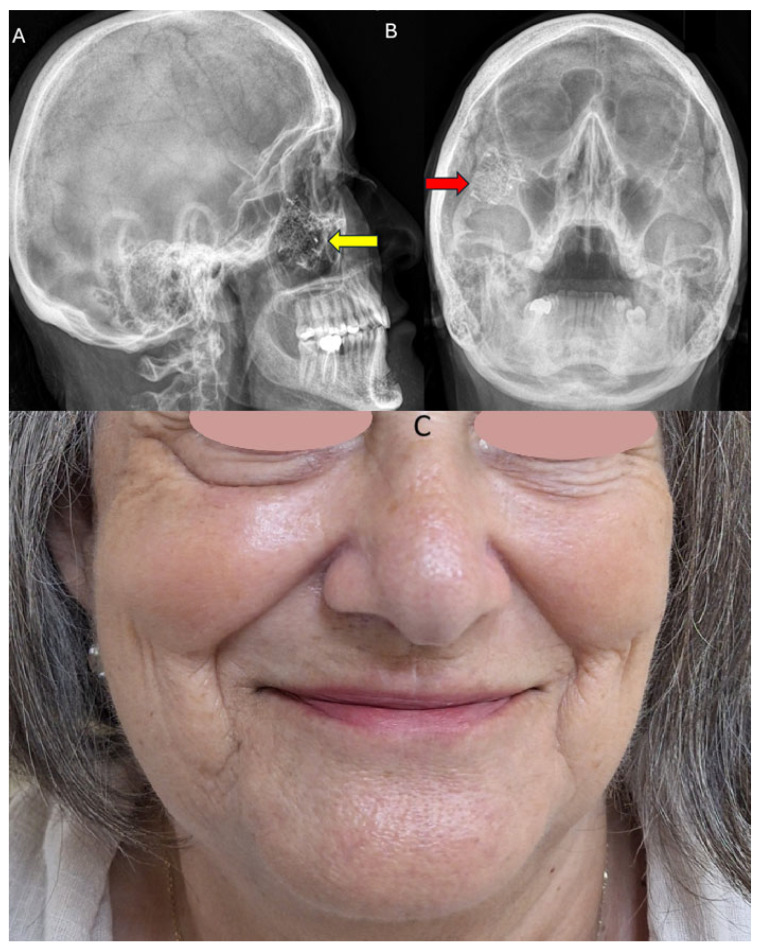
Control post-operative lateral ceph (yellow arrow) radiograph revealed proper mesh alignment. The maxillary sinus is radiolucent and without signs of infection, indicating that it remained intact during the operation (**A**). Waters’ radiograph revealed a very good bone symmetry and zygomatico-malar prominence position. The titanium mesh can be noticed in the proper position in the body of the right zygomatic bone, transfixed in the infraorbital rim with titanium screws (Modus 2.0, Medartis, Basel, Switzerland) (red arrow) (**B**). The final result after surgery is excellent. The patient was examined 5 months after surgery in the outpatient clinic. The cosmetic result is outstanding, and the scar is barely visible. The function of the infraorbital nerve was maintained, and midface anatomy was restored to normal without any secondary deficits. The scar is well hidden in the facial wrinkles in relaxed skin tension lines (RSTLs) (**C**). Facial nerve function is maintained, and facial expression remains the same. The patient remains under clinical observation. A good CT examination, followed by PSI-guided 3D-printed models, grants a safe surgery with good outcomes.

## Data Availability

The datasets used and/or analyzed during the current study are available from the corresponding author upon reasonable request.
